# Evaluation of the forum theatre approach for public engagement around antibiotic use in Myanmar

**DOI:** 10.1371/journal.pone.0235625

**Published:** 2020-07-09

**Authors:** Myo Maung Maung Swe, Phyu Hnin Hlaing, Aung Pyae Phyo, Htet Htet Aung, Frank Smithuis, Elizabeth A. Ashley, Phaik Yeong Cheah

**Affiliations:** 1 Myanmar Oxford Clinical Research Unit (MOCRU), Yangon, Myanmar; 2 Nuffield Department of Clinical Medicine, Centre for Tropical Medicine and Global Health, University of Oxford, Oxford, United Kingdom; 3 Mahidol-Oxford Tropical Medicine Research Unit, Faculty of Tropical Medicine, Mahidol University, Bangkok, Thailand; 4 Lao-Oxford-Mahosot Hospital Wellcome Trust Research Unit (LOMWRU), Vientiane, Laos; Ravensburg-Weingarten University of Applied Sciences, GERMANY

## Abstract

**Introduction:**

The risk of emergence and spread of antibiotic resistance is high in Southeast Asian countries and various strategies are being used to raise awareness about appropriate antibiotic use and antibiotic resistance within communities. Public engagement in science has not been widely practised in Myanmar. We describe the use of a forum theatre to engage with the community about antibiotic use.

**Methods:**

The engagement activities took place in a peri-urban township in Yangon, Myanmar. Five preliminary story gathering workshops with the community were carried out to develop scripts and songs for the forum theatre. After that, we organised forum theatre plays between September and October 2018. Following each play we provided four simple key messages based on WHO’s world antibiotic awareness week advocacy materials; 1) Antibiotics are medicines used to treat bacterial infections 2) Antibiotics are not useful for coughs and colds 3) Never use leftover antibiotics or share antibiotics with others 4) Prevent infections by regularly washing hands, preparing food hygienically, avoiding close contact with sick people, and keeping vaccinations up to date. We evaluated the engagement activities by conducting focus group discussions (FGD) with audience members.

**Results:**

Ten forum theatre plays were performed on two topics; “Fever and antibiotics” and “Mixed medicines”, reaching 1175 community members. Four themes emerged from our thematic analysis: 1) Knowledge dissemination, 2) Enjoyment and fun, 3) Willingness to support and recommendations for future engagement activities and 4) Preference over traditional methods of health education. We found improvement of antibiotic related knowledge and enjoyment among audience who were also willing to support future engagement activities and preferred forum theatre approach over formal health talks.

**Conclusions:**

We conclude that forum theatre is an effective innovative approach to engage and disseminate knowledge on appropriate use of antibiotics with the community in a participatory way.

## Introduction

Antibiotics are losing their efficacy at an alarming rate due to the emergence and rapid spread of antibiotic resistant bacteria. Inappropriate use of antibiotics is accelerating this process. Global antibiotic consumption has increased by 65% from 2000 to 2015 driven mainly by use in low- and middle-income countries (LMICs), with increased use of last resort antibiotics [1]. Without intervention, it is projected that antibiotic consumption will increase 200% by 2030 compared to 2015 figures [1].

Myanmar is a lower middle income country in the South East Asia Region with a high risk of emergence and development of antibiotic resistance in humans [2]. A situation analysis reported that 87% of patients with upper respiratory tract infections in primary care in Myanmar received an antibiotic prescription [3]. A fever study in Myanmar reported that 69% of febrile patients in the outpatient department and 41% of general patients received an antibiotic in primary care clinics [4]. Although Myanmar has established an Antimicrobial Resistance (AMR) working group to develop a National Action Plan which was released in 2017, national policy and regulation for antimicrobial use are still in the early stages of development [5]. Risk of spread of antibiotic resistance in Myanmar is high and a cross sectional hospital based study done in Yangon in 2016–2017 showed that 68% of the isolates were found to be multidrug resistant organisms (resistant to more than three classes of antimicrobial drugs) and 8% of the Gram negative isolates were found to be carbapenemase producers [6]. Drug shop surveys done in two cities of Myanmar found that antibiotics such as amoxicillin, cotrimoxazole, erythromycin, metronidazole, tinidazole, ciprofloxacin, norfloxacin and neomycin are sold without prescription [7, 8].

In 2015, the WHO endorsed a global action plan to tackle AMR and one of the key recommended strategies is to improve awareness and understanding of antimicrobial resistance through effective communication, education and training [9]. In the light of over the counter (OTC) availability of antibiotics in Myanmar, it is important to understand how the community make decisions about antibiotic use for febrile illness. A recent study exploring patients’ conception of illness and medicine use conducted in Chiang Rai, Thailand and Yangon, Myanmar showed that antibiotic intake was likely to be influenced by their perception on the nature of illness [10]. We designed a public engagement project using a forum theatre technique to better understand antibiotic use for febrile illness and to raise awareness about appropriate use of antibiotics in the community.

Forum theatre is part of a broader “Theatre of the Oppressed” technique developed by a Brazilian theatre director, Augusto Boal, in the 1970s [11]. In forum theatre, actors perform twice with some oppressed characters in the plays. The first play usually ends up with an unsolved problem or tragic ending. During the replay, the audience members (or ‘spect-actors’) are invited on stage to take the role of oppressed characters and are allowed to solve the problems faced by the characters. Forum theatre needs good facilitation to ensure the smooth flow of the play and active participation by audience members [11]. Forum theatre techniques have been used in different areas of biomedical science including research and clinical trials [12], health promotion [13], health education [14] and training of medical professionals [15–17]. We chose forum theatre because it is interactive, does not rely on literacy and encourages audience members to share their knowledge with others.

The aims of this engagement project were to understand antibiotic use for febrile illness and to raise awareness about appropriate use of antibiotics in the community.

We evaluated the engagement activities in order to 1) assess whether the engagement fulfilled the aims of the project, 2) identify knowledge disseminated through the engagement, and 3) provide feedback to the organizers for future engagement activities. This paper describes the forum theatre project and reports the results of our evaluation.

## Materials and methods

We collaborated with an established local theatre group, “Arts for All”, in the engagement project, who received forum theatre training from Pan Arts international in UK. “Arts for All” have been using forum theatre since 2011 to raise awareness and encourage behavioural change related to health issues such as reproductive health and HIV/ AIDS, and justice issues in Myanmar (https://www.britishcouncil.org.mm/programmes/arts/human-drama). We sought permission from the Yangon region health department to conduct the project. Detailed plans and timeline of the project (Table 1) were discussed with general administrative authorities in the project area.

**Table 1:** Timeline of activities.

No	Activities	Timeline
1	Consultative meetings with health and administrative authorities	Apr—May 2018
2	Story-gathering workshops	July—Aug 2018
3	Development of scripts and songs	July—Aug 2018
4	Forum theatre plays	Sep—Oct 2018
5	Evaluation	Sep—Oct 2018

### Project location

We carried out the project in Hlaingtharya (HTY) Township located in the western part of Yangon, one of the biggest townships in the country with an estimated population of 687,000 as per 2014 Myanmar population and housing census. We selected five locations to perform the engagement activities after discussion with township administrative authorities. These locations were selected because they were overcrowded areas with poor living conditions and insufficient water and sanitation conditions. These factors are believed to contribute to a high risk of infectious diseases. Most residents living in these areas are manual and factory workers with below average socio-economic status.

### Design of the forum theatre performance

#### Story-gathering workshops

As forum theatre techniques use real life scenarios, we arranged story gathering workshops to identify common health issues related to participants’ experience of febrile illness. With the help of community leaders, we invited community members from diverse backgrounds including housewives, manual workers and members of civil society organizations. Questions related to treatment-seeking for febrile illness, access to antibiotics and use of mixed medicine packs [a bag which contains a mixture of different medicines such as analgesics, antibiotics and sometimes steroids that is available without prescriptions in local shops] were asked during the workshop. Between July and August 2018, we carried out five story gathering workshops in the areas selected. Each workshop lasted approximately one hour and was attended by between 25 to 35 participants. The issues raised during the workshops were prioritized by the consensus method. Two issues related to febrile illness: “Fever and antibiotics” and “Mixed medicines” were chosen as the themes for the forum theatre play.

#### Storylines

The theatre group together with the project team developed songs and plots for the forum theatre plays based on the two issues identified during the workshops. We included colloquial words in the script to provide the audience with a sense of familiarity. The storylines centred on a sick person (a central character) who took antibiotics without prescription and “mixed medicine” purchased from local shops. Family members, neighbours and community members tried to persuade the sick person not to take antibiotics without prescription or the “mixed medicine” in different scenes of the play. Both storylines ended with the sick person being hospitalized due to side effects of antibiotics and failure to seek appropriate treatment from health professionals.

#### Forum theatre plays

The theatre group was made up of 10 youth actors of both genders and led by a project manager. Each play was performed by seven actors over five or six scenes and lasted approximately 45–60 minutes including the replay assisted by a facilitator. The facilitator explained the nature of the play to the audience at the start and encouraged them to participate actively in the replay. During the replay, any audience members who had different alternative suggestions were invited to the stage to take the role of the actors. In the replay, there were alternative endings that included successfully convincing the sick person to seek appropriate care from health professionals instead of self-medication with antibiotics and “mixed medicine”. Two to four audience members actively participated in each replay (Link: https://www.youtube.com/watch?v=4dC4PLygsHM). Four key messages were delivered after each play. These were based on the WHO’s World Antibiotic Awareness week advocacy materials: 1) Antibiotics are medicines used to treat bacterial infections 2) Antibiotics are not useful for coughs and colds 3) Never use leftover antibiotics or share antibiotics with others 4) Prevent infections by regularly washing hands, preparing food hygienically, avoiding close contact with sick people, and keeping vaccinations up to date. We conducted pre and post focus group discussions (FGD) on the same day.

### Evaluation

We collected audience numbers of each performance, and qualitative data to capture perspectives of the people we engaged. We used four main evaluation questions to guide evaluation process and establish relevant data collection methods to achieve the objectives. Evaluation questions and data collection methods were outlined in Table 2.

**Table 2:** Evaluation questions and data collection tools.

Evaluation Questions	Data collection
1) What is the community’s understanding about the role of antibiotics to treat febrile illness?	Pre and post-performance FGDs, intervention action of audience members in the replay
2) Have the engagement activities improved the community understanding of antibiotics and their appropriate usage?	Pre and post-performance FGDs
3) What did the community think about the forum theatre approach for public engagement?	Pre and post-performance FGDs
4) What were the challenges during the process of conducting these activities?	Field notes and reflective analysis throughout the engagement process with different level of authorities

#### Qualitative data collection

Qualitative interviews were led by an experienced local qualitative researcher (PHH) assisted by a research assistant (HHA). The lead researcher is a health professional with several years’ experience in conducting qualitative research and familiar with the local context.

*Focus Group Discussions (FGDs)*. Pre and post-performance FGDs were conducted for four out of ten plays in total. Three questions were included in the pre-performance FGD topic guide: i) What are antibiotics? ii) What are they used for? iii) Does all fever need antibiotics? In the post-performance FGD topic guide, we added five more questions in order to capture feedback and suggestions on the forum theatre initiative. The guideline questions are outlined in Table 3.

**Table 3:** Guideline questions used in pre and post-performance focus group discussions.

Pre-performance questions	Post-performance questions
1. What are antibiotics?	1. What are antibiotics?
2. What are they used for?	2. What are they used for?
3. Does all fever need antibiotics?	3. Does all fever need antibiotics?
	4. What did you learn from the performances?
	5. What do you think about the replays with audience interventions?
	6. If you had a chance to intervene, would you have done it and why?
	7. What are the things you dislike about the performance?
	8. Do you have any general recommendations for the performance/project (place, timing, etc.)?

*FGD respondents*. Forty individuals participated in four pre and post FGDs. They were selected based on their availability for discussion and willingness to participate. Pre and post FGDs were conducted with the same participants. In addition, audience members who actively participated during the replays were invited to join the post-performance FGDs.

*Procedures*.All FGDs were conducted in the same location as the plays. Discussions were conducted in Burmese language (official language in Myanmar) and the sessions were audiotaped. Before the start of an FGD, a brief explanation regarding the general purpose of the engagement, approximate duration needed and the importance of keeping confidentiality within the group were explained to the participants. The questions outlined in Table 3 were used to guide the discussions. Each FGD lasted between 40 to 60 minutes.

*Data analysis*. Recorded audio files were transcribed verbatim. All the transcripts were translated to English which were analysed using content analysis. The content analysis included identifying categories, comparing and contrasting the various major and minor categories, reviewing the categories, and finally returning to the original transcripts to ensure all the information that needed to be categorized had been done. The triangulation method established with the project team members to check the integrity of the inferences drawn.

#### Audience numbers

The number of audience members who attended forum theatre play and those who intervened during the replays were recorded in attendance sheets.

### Ethical considerations

We obtained permission from the Yangon regional health department and Hlaingtharya general administrative department to conduct the engagement project. We discussed evaluation of the project with the Oxford Tropical Research Ethics Committee (OxTREC) who granted a waiver from ethics committee review. We obtained verbal consent from those who were involved in FGDs. Participant names, addresses were not recorded and all other information that might identify participants were removed from the transcripts.

## Results

We organised ten forum theatre plays on two topics; “Fever and antibiotics” and “Mixed medicines”, reaching 1175 community members between September and October 2018. A total of 36 community members intervened during the replays. Details of plays and audience numbers are shown in Table 4.

**Table 4:** Details of forum theatre plays, topics, numbers attended and intervened during the replay.

No	Date	Location	Topic of plays	No. of Audience members	No. audience members who intervened in the replay
1	8-Sep-18	HTY Ward 1	Fever and antibiotics	106	4
2	9-Sep-18	HTY Ward-Shwe Lin Pan	Fever and antibiotics	110	4
3	22-Sep-18	HTY Ward 6	Mixed medicines	126	4
4	23-Sep-18	HTY Ward 1	Mixed medicines	136	4
5	23-Sep-18	HTY Ward-Shwe Lin Pan	Mixed medicines	83	4
6	6-Oct-18	HTY Ward 6	Mixed medicines	104	3
7	7-Oct-18	HTY Ward 5	Fever and antibiotics	132	3
8	14-Oct-18	HTY Ward 5	Mixed medicines	132	2
9	18-Oct-18	HTY Ward 19	Fever and antibiotics	123	4
10	18-Oct-18	HTY Ward 19	Mixed medicines	123	4
	Total	5 wards	2 themes	1175	36

The qualitative data derived from the FGDs are presented according to four themes: 1) Knowledge dissemination 2) Enjoyment and fun, 3) Willingness to support engagement activities and recommendation for future activities, 4) Preference of forum theatre over traditional methods of health education delivery e.g. health talks

1 Knowledge dissemination

Before the forum theatre performances, we noted that most participants had just heard the term “antibiotics” but they did not know exactly what antibiotics were and their usage. Some participants gave their opinion on what antibiotics were based on their experiences, Consider these quotes:

“I know antibiotic such as amoxicillin and they are used when fever is very high” (FGD 2)

“Antibiotics needed to be taken when you get dog bites, or attacked by any poisonous animals like snakes” (FGD 1)

“Antibiotics can also be used when we have pains in arms and legs with high fever” (FGD 3)

“I heard antibiotics are used for diarrhoea” (FGD 4)

“I know that we use antibiotics to stop spreading the diseases” (FGD 4)

“We can use antibiotics to prevent seasonal flu” (FGD 1)

“I think antibiotics are used for TB (tuberculosis) and I don’t think we should use for other diseases” (FGD 3)

“I also heard that antibiotics are used to help other medicine’s effects” (FGD 2)

“Different antibiotics need to be used for children. They must not be the same with antibiotics for adults” (FGD 2)

Some of the above quotes illustrate correct understanding of antibiotics, while others demonstrate some common misunderstandings of their uses of antibiotics.

After watching the forum theatre, most FGD participants mentioned that antibiotics are medicine used to kill bacteria: ‘***poe tat say***’ in Burmese language.

“Now I know antibiotics are medicines which kill bacteria” (FGD 2)

“Now I know that antibiotics are used to kill bacteria and we shouldn’t take them every time we have symptoms like sneezing and headache” (FGD 1)

“We learnt that we shouldn’t take mixed medicines on our own without doctors’ prescription” (FGD 2)

“We should not take antibiotics and analgesic all the time” (FGD 3)

“And we learned that being superstitious is not good” (FGD 2)

“We should stop people from using antibiotics carelessly” (FGD 1)

“We realized that we should go to clinics/hospitals rather we go the drug store and get random drugs” (FGD 1)

2 Enjoyment and fun

From our observations and FGDs, it was clear that audience members enjoyed the plays. FGD participants also commented on specific parts of the plays they enjoyed and their overall satisfaction with the project.

“I really enjoyed the part of the play where May Thu (a character in the play) resisted to go to the hospital” (FGD 4)

“I like that kinds of short plays. They are interesting and very informative” (Participant from FGD 2)

“Kids are happy as well” (FGD 1)

3 Willingness to support engagement activities and recommendations for future activities

When participants were asked about their satisfaction and potential support for future events, many of them expressed their willingness to support similar kinds of events and wanted to see more on different health topics.

“We will support if you are doing more project like this” (Participant from FGD 2)

“We would like to have more plays on TB (tuberculosis), Hepatitis C and also women topics like using contraceptives” (FGD 2)

“For next time, please do health education talk on TB, we also have a lot of immigrants, who need to see this kind of show” (FGD 2)

“We also want to see plays on drugs use, especially for kids. We have kids who use drugs in school, we don’t know what we should do about that” (FGD 2)

They also had some practical suggestions for future engagement work.

“Performance overall was good but we think the performers could have paid more attention the settings to look more realistic e.g. the pharmacy, they could have built a small shop on stage or something similar a separate dispensing room” (FGD 2)

“Regarding the timing, we prefer Sundays because we have to go work on Saturday as well” (FGD 4)

4 Preference of forum theatre over traditional methods of health education delivery (e.g. talks)

During the FGD, participants also compared forum theatre with more formal health education approaches.

“We prefer this kind of health education compared with the formal health talks. This one is more interesting” (FGD 2)

“And this kind of performance is more fun and enjoyable and also more informative” (FGD 2)

In addition to data derived from the FGDs, we obtained additional insights from actions by participants during the replay. By witnessing actions of audience members who intervened during replay, we noted and learnt the following:

1. Most intervening audience members did not appear to know why taking antibiotics without prescription was inappropriate. They only advised to consult with medical doctors for febrile illness.

2. Family hierarchy played an important role because the intervening person who took the role of a junior family member faced challenges and was almost unsuccessful when trying to convince senior family members not to take antibiotics without prescription.

3. Audience members who took the role of neighbours were generally willing to offer financial help in case the sick person had financial hardship to go to general practitioners or hospital.

### Challenges during the process of engagement and lessons learnt

A combination of poor drainage systems in the selected areas and heavy rain during the monsoon season made us postpone and reschedule forum theatre plays several times. In addition, the rain discouraged the audience from coming to the plays. Most of the places for the plays provided by the administrative authorities were Buddhist religious buildings which may have excluded community members from other religions. We were only able to conduct engagement activities at weekends as most residents were at work during the week. We learnt that involvement and active participation of the administrative authorities and community (including community leaders and members) was vital for the smooth running of all engagement activities. We invited key community members including ward administrative officers and community leaders to our forum theatre plays.

## Discussions

Although the number of public engagement activities on health and research has been growing in the past decades, literature on the evaluation and impact of these activities is limited [18].

We evaluated our engagement activities not only to provide insights into potential challenges for the organizers during the different phases of the engagement but also to capture the information conveyed and the participants’ experiences of the event. We evaluated forum theatre public engagement by employing qualitative methods (FGDs and observations) in addition to documenting the number of attendees and participants who took part in the replay.

There has been an increase in public engagement activities in science and research in Southeast Asia in recent years, for example “Pint of Science” and “Science Cafes” events have increased in Thailand, Cambodia and Laos [19–21]. Public engagement activities have not been practised widely in Myanmar to date [22]. Drama has been used in neighbouring countries for engagement activities although not employing the forum theatre technique [23–26].

Formal health education activities are quite common within local communities, but communication is usually one way, and two-way interactions are minimal. In our activity we invited community members to get involved in all stages of engagement activities from story gathering workshops to replays and evaluation. Evaluation of a community engagement drama project in Cambodia also highlighted the importance of community involvement throughout the engagement activities [24].

Forum theatre is an audio-visual interactive type of activity in which participants have the ability to make changes on stage to reach a better outcome which encourages them to use reasoning and take decisions by playing a role to correct the undesirable behaviours. Findings from our evaluation showed that all the participants enjoyed the plays and they perceived that they had gained knowledge about antibiotics.

Our engagement activities allowed the audience to express their own ideas and experiences related to the topics of engagement while learning new knowledge. It also gave a unique and creative experience to the audience members as they were able to take the role of actors during the replay. The engagement also empowered the audience by giving them the sense that they were able to influence the storyline of the performance to reach a better outcome. These findings are consistent with the findings of another study where a theatre-based educational initiative was used to raise awareness and knowledge about eclampsia in rural areas of Bangladesh [14]. We expect that our findings might be generalizable to peri-urban slum areas of other low- and middle- income countries in terms of active community participation, interactive engagement, knowledge improvement and empowerment of the community. However, we recognise that the characteristic of forum theatre group plays an important role in the project. Our theatre group consisted of young enthusiastic actors, which may have been key to the active participation by the community.

Post-forum theatre FGDs showed improvements in understanding on the appropriate usage of antibiotics but that might not reflect in-depth understanding. A study to improve flu vaccination among health care workers in a university hospital showed that forum theatre raised the awareness and had a positive impact on the message delivered [13]. Participants also expressed their willingness to support similar kinds of events in their community. From the number of attendees at each event, we conclude that the preparatory phases were successful in establishing effective strategies to communicate with both authorities and local communities about health and research topics. Through audience participation in the replay, we learnt about how the community solved problems related to antibiotic use. Making a change at different levels of the community such as individuals within families, neighbours and the wider community could lead to lasting changes towards appropriate antibiotic use.

Our paper has several strengths. To the best of our knowledge this is the first paper that reports the use of forum theatre around antibiotic use in Myanmar and its evaluation. We used qualitative method for evaluation to get better understanding of the effectiveness of our engagement activities. The number of attendees in each performance reflected the people reached by our engagement activities.

Our study also had some limitations. In this pilot engagement project, we performed a one-time evaluation on the same day as the plays. It only allowed us to evaluate knowledge gained and perceptions/ opinions immediately after the forum theatre event. We did not evaluate retention of knowledge or behavioural changes after the engagement activities. In addition, our project was conducted in peri urban slum areas, but the majority of the population in Myanmar resides in rural areas Future engagement activities should include hard-to-reach communities in both urban and rural settings [27]. Future engagement activities with longitudinal evaluation at different time points could allow assessment of retention of knowledge and changes in practices over time.

## Conclusions

We conclude that forum theatre could be used as a fun and engaging way of conducting public engagement around health. Our project aims which were to understand antibiotic use for febrile illness and to raise awareness about appropriate use of antibiotics in the community, were met. It allowed audiences not only to gain health knowledge related to appropriate antibiotic use but also to express their ways of solving issues around antibiotics use by interacting with actors and other audience members. At the same time, our team were able to learn what the communities understand about antibiotics and how they make decisions of using antibiotics in real-life scenarios as well as their ways of tackling the issues related to the themes performed. To sum up, forum theatre could be an innovative way to engage and promote health related knowledge with the community.
